# Improving the Wear-Resistance of BT22 Titanium Alloy by Forming Nano-Cellular Topography via Laser-Thermochemical Processing

**DOI:** 10.3390/ma16113900

**Published:** 2023-05-23

**Authors:** Oleksandr Tisov, Alina Yurchuk, Mykhaylo Pashechko, Iryna Pohreliuk, Dariusz Chocyk, Myroslav Kindrachuk

**Affiliations:** 1 School of Aerospace Engineering, Xi’an Jiaotong University, West Xianning Road 28, Xi’an 710049, China; 2 Department of Air Transport Organization, Faculty of Transport, Management and Logistics, National Aviation University, Lubomyra Huzara Ave. 1, 03058 Kyiv, Ukraine; alina.yurchuk@npp.nau.edu.ua; 3 Department of Fundamentals of Technology, Fundamentals of Technology Faculty, Lublin University of Technology, Nadbystrzycka Str. 36, 20-618 Lublin, Poland; mpashechko@hotmail.com; 4 Karpenko Physico-Mechanical Institute of the NAS of Ukraine, Naukova Str. 5, 79060 Lviv, Ukraine; irynapohrelyuk@gmail.com; 5 Department of Applied Physics, Faculty of Mechanical Engineering, Lublin University of Technology, Nadbystrzycka Str. 36, 20-618 Lublin, Poland; d.chocyk@pollub.pl; 6 Aerospace Faculty, National Aviation University, Lubomyra Huzara Ave. 1, 03058 Kyiv, Ukraine; myroslav.kindrachuk@npp.nau.edu.ua

**Keywords:** selective laser processing, nitriding, two-phase titanium alloy, BT22 titanium alloy, wear-resistance

## Abstract

This paper studies the microstructure, phase composition and tribological response of BT22 bimodal titanium alloy samples, which were selectively laser-processed before nitriding. Laser power was selected to obtain a maximum temperature just a little above the α↔β transus point. This allows for the formation of a nano-fine cell-type microstructure. The average grain size of the nitrided layer obtained in this study was 300–400 nm, and 30–100 nm for some smaller cells. The width of the “microchannels” between some of them was 2–5 nm. This microstructure was detected on both the intact surface and the wear track. XRD tests proved the prevailing formation of Ti_2_N. The thickness of the nitride layer was 15–20 μm between the laser spots, and 50 μm below them, with a maximum surface hardness of 1190 HV_0.01_. Microstructure analyses revealed nitrogen diffusion along the grain boundaries. Tribological studies were performed using a PoD tribometer in dry sliding conditions, with a counterpart fabricated from untreated titanium alloy BT22. The comparative wear test indicates the superiority of the laser+nitrided alloy over the one that was only nitrided: the weight loss was 28% lower, with a 16% decrease in the coefficient of friction. The predominant wear mechanism of the nitrided sample was determined to be micro-abrasive wear accompanied by delamination, while that of the laser+nitrided sample was micro-abrasive wear. The cellular microstructure of the nitrided layer obtained after the combined laser-thermochemical processing helps to withstand substrate deformations and provide better wear-resistance.

## 1. Introduction

Titanium alloys are famous for their excellent mechanical performance and for being lightweight [1]. Moreover, they mostly do not corrode [2] and can work well in human organisms [3,4,5,6]. Nevertheless, their significant drawbacks are higher costs and low wear-resistance. Therefore, for good performance, their surface should be coated with a protective or barrier layer [7,8]. For this purpose, many methods have been developed. The most widely used types of coatings are diffusion [9,10] and overlay [11,12].

Among titanium alloys, those that have a bimodal microstructure have high toughness and ductility, and they may be processed by heat treatment or used in the annealed condition. Many of these alloys, such as 5553, are well-known and studied and are often reported in the literature [13,14]. As studied in this paper, alloy BT22, a modification of Ti-5553, has been tested by [15,16,17,18]. Its mechanical properties depend on the size and structure of the α-phase particles and the α/β ratio. This ratio and the morphology of the α-phase may be altered by heat treatment. Thermochemical processing with oxygen and nitrogen also increases the amount of α-phase on the surface, which improves its hardness and wear-resistance [15].

Plasma nitriding has been intensively used for the nitriding of titanium alloys, especially for biomedical applications [6]. Here, the wear-resistance and being friendly to the live tissue is more important than strength and fatigue-resistance. The use of novel gas mixtures during combined plasma-gas nitriding allows us to achieve hard surfaces (900–1100 HV_0.02_, [19]) at a temperature of 600 °C. This was achieved by the formation of both titanium nitrides and hydrides. The significant drawback of this method was the low thickness of the nitride layer: 1–3 μm. Plasma nitriding at 730 °C for 4 h allowed us to obtain another layer of 1.8–3.2 μm. The top-surface was composed of nano-grained nitrides [20,21], which is a promising treatment for implants.

Recently, laser processing has been widely used for the nitriding of titanium alloys. It helps to modify the surface layer and supply the required amount of saturating elements to the surface in a short time [22]. The novel plasma-enhanced pulsed-laser deposition method [23] allows for the acquisition of a 100 nm diffusion layer in 1 h. It has good properties, but in our opinion, it is too thin for tribological application, even for biomedical systems.

Laser-assisted gas nitriding also has an essential drawback: its high-power consumption [24] entails a much greater energy requirement (1–5 kW vs. 400–650 W [19]) for processing. The benefit of titanium laser nitriding is that it treats only the surface where the beam is focused, and it may be used selectively to create any imaginable pattern and broad temperature ranges within one processing installation [25,26]. Laser processing may be used to perform surface texturing for lubricant film regeneration; laser power was used to create patterns, and after that, to activate nitrogen diffusion and refine microstructure [27]. While laser nitriding involves surface remelting and requires the precise control of saturation parameters, it is also hard to process internal surfaces via this method (e.g., holes for the insertion of bronze landing gear bushings). Obtaining a thick coating is not always possible [28], though in other papers [29] the reported nitride layer thickness was 56 μm.

All the nitriding methods have a few things in common: high temperature and a controlled atmosphere containing nitrogen. Some of them, such as plasma nitriding, may cause the burning of the edges or increase the surface roughness and diffusion of unwanted gases. The simplest, most studied, and most predictable nitriding method is gas nitriding [10,30,31,32]. Its drawback is its long duration. The other durable and high-temperature process is titanium heat treatment, which is a two-step aging process [14,15,16,17]. So, it is essential to combine them, which was previously studied by the authors in [33]. As a result, a significant decrease in the friction coefficient and wear-resistance of dry sliding wear was achieved. It was also reported in [25,34] that laser processing may form the cell-like microstructure that could significantly improve alloy tribological output and serve to recover the lubrication film. The combination of nitriding and post-selective laser treatment also improves the alloy tribological and biomedical performance [35].

Surface-modified titanium alloys are widely used in the fields of medicine [3,4,5,6] and engineering [7,8,9,10,11]. Besides high strength, their tribological performance is of primary importance. The urgent task of improving the wear-resistance of the high-strength bimodal titanium alloy BT22 may be solved through the thermochemical treatment of the selectively laser-processed surface. This treatment changes the alloy’s microstructure and creates a mechanical gradient. The thermochemical processing of laser-treated material may be effectively performed during a standard heat treatment, where controlled nitrogen flow is used instead of a vacuum. In this study, the authors also study the cell-like microstructure formed by two-step technological process. The first step—selective laser treatment—refines the microstructure for the promotion of nitrogen diffusion; the second step involves duplex aging and nitriding treatment.

## 2. Materials and Methods

Discs of the two-phase (α+β) titanium alloy BT22 (Ti-5Al-4.75Mo-4.75V-1Cr-1Fe) [13] (see Table 1) were cut from a Ø55 mm bar using a Beta-300 Pro automatic cutting machine (Trojan, Suzhou, China). The alloy contained both α- and β-stabilizing elements at nearly equal concentrations: K_β~_1.0 [16]. Disc thickness was 5 mm, which provided enough stiffness during the wear tests. Pin counterparts with a diameter of 8 mm were fabricated from the Ø12 BT22 bars by turning them on a lathe to obtain the required size. The samples and counterparts were ground to the roughness of R_a_ 0.5 μm using a M250 surface grinder (BMD, Suzhou, Jiangsu, China).

Before the nitriding, and in order to remove possible surface deposits, the discs were cleaned in a K360HTDP sonicator (Anonkia, Zhangmutou, Guangdong, China) for 5 min. The pin was used in supply condition, and the sample was laser-treated and nitrided. We used a DY 044 laser (Rofin Sinar, Hamburg, Germany) with a focused spot diameter of 2.5 mm for the laser treatment. The treatment was selective, with a distance of 2.5 mm between the laser focusing centers and with each circle touching four neighboring circles. The nitrided alloy will be referred to as Sample 1 and the alloy subjected to the selective laser processing and post-nitriding as Sample 2.

**Figure 1:**
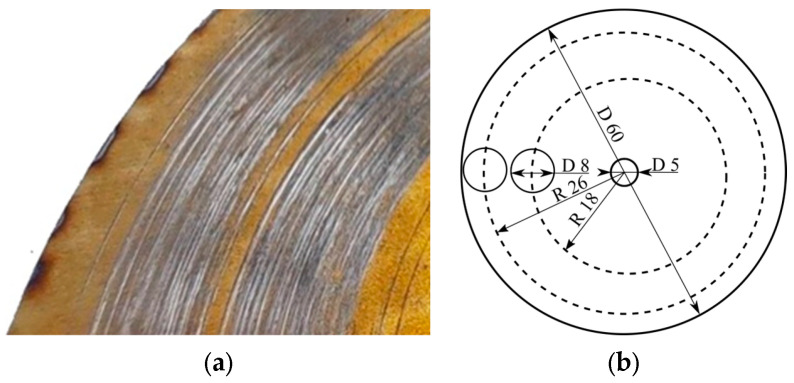
Positioning of pins on the disc surface: (**a**)—tested surface, (**b**)—dimensions and position of the pins over the disc.

The laser power density was 0.4 GW/cm^2^, which was the density needed to cause a surface temperature of 880–900 °C, which is above the α-β transus point. This treatment should refine the structure of the surface. For the treatment, the discs were placed in a vacuum furnace and preheated. After this, the vacuum was replaced with nitrogen, and the treatment proceeded in two steps under thermostatic conditions. As it was mentioned above, the BT22 alloy may be used in annealed or age-hardened conditions. For this study, we selected the second case as it is more durable; it would also allow us more time for thermochemical processing and have a less detrimental effect on the structure obtained due to laser treatment. Figure 1a,b shows the geometry and positioning of the pin and disc during the wear tests. Both sides of the sample were identically treated; one disc was used for four tests.

**Figure 2:**
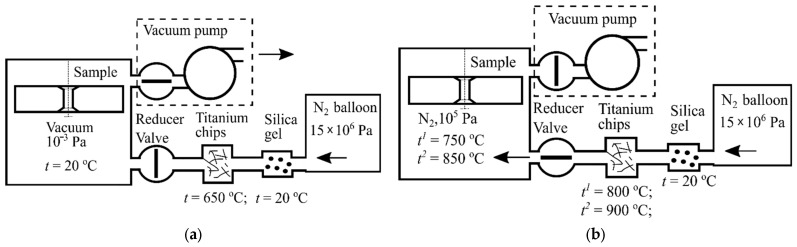
The layout of the nitriding furnace: (**a**)—furnace with the sample inside is ready for thermochemical processing; (**b**)—double-step aging and nitriding process; t—the temperature of furnace sections with evacuated air, prepared for thermochemical processing, t^1^—temperatures of furnace sections during the first (4 h) stage of heat treatment, t^2^—temperatures of furnace sections during the second (1 h) stage of heat treatment.

Thermochemical processing was performed during the standard aging procedure for the alloy BT22. A self-designed vacuum furnace (Figure 2) was equipped with an additional nitrogen supply line as it allowed for the use of the so-called industrial-quality gas taken from the air at nitrogen extraction plants and stored in balloons under the pressure of 15 MPa.

**Figure 3:**
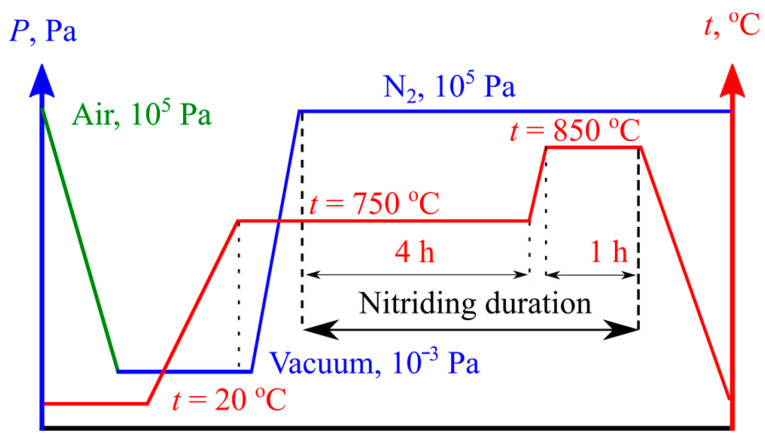
Titanium BT22 aging-nitriding temperature and pressure graphs.

As it could contain impurity gasses and solid contaminants, it was filtered at the exit of the gas store. The dryer box was filled with silica gel. The preheated chips of the chemically pure titanium effectively retained oxygen and allowed for the control of its concentration in the reaction chamber inside the furnace. To create the required conditions, the reducer valve was closed, and the air was evacuated. Nitrogen pressure could be controlled in a broad range, which also allowed for the creation of a dynamic gas flow during the processing; both “in” and “out” valves were partially opened, and a constant nitrogen flow was passed around the sample. The furnace capacity allowed for the processing of several samples without any influence on each other. For this study, we prepared the nitrided and laser+nitrided samples simultaneously so that their gas/thermodynamic conditions would be identical. A two-step aging process is usually used for the alloy BT22 in order to obtain high strength. The temperature and pressure graphs for the nitriding are reported in Figure 3.

The furnace was firstly loaded with the samples. They were hung in order to prevent them from touching each other. When the vacuum pressure decreased to 10^−3^ Pa, we gradually increased the temperature to avoid thermal shocks, which could occur on the edges (see Figure 1a). When the temperature inside the chamber and of the sample reached equilibrium, we allowed the nitrogen to flow in until the pressure inside reached P_N2_ = 10^5^ Pa. The silica gel dryer reduced the possible moisture, while the CPT chips preheated to t^1^ = 800 °C reduced the oxygen content. The first aging period at 750 °C lasted for 4 h, and it was followed by the second period at a temperature of 850 °C for 1 h. The task of the last stage was to intensify the formation of nitrides in the surface layer. The total nitriding duration was 5 h.

The alloy microstructure of normal sections was studied before and after the nitriding. As an etchant, we used a standard Kroll solution: 5% of HNO_3_, 3% of HF, and 92% of H_2_O.

The microstructure of the modified layers of the titanium alloys was examined using an Axio Scope A1 light microscope (Zeiss, Oberkochen, Germany), and to measure the surface roughness, a Keyence VK9710 laser confocal microscope was used. The wear track morphology was observed using a SEM-FIB DualBeam Scios 2 (Thermofisher Scientific, Waltham, MA, USA). The Vickers microhardness was measured with a PMT-3M instrument (LOMO, sankt-peterburg, rf) under an indenter load of 50 and 10 g (HV_0.05_ and HV_0.01_). The thickness of the nitride layer was determined through metallographic and hardness profile methods [32,36,37].

We used the HTH 1000 precision tribometer (Anton Paar, Buchs, Switzerland) for the wear test, at a clamping pressure of 0.2 MPa. The sliding speed was 0.5 m/s, and the sliding distance was 300 m. In Figure 1b, each of the two sides of the sample was tested two times. For the first test, the pin installation radius was 26 mm, and for the second, it was 18 mm. Then, the sample was upset, and two new tests were carried out. To keep the sliding speed constant, the first test on the outer raceway was conducted at 184 rpm, and the second on the inner raceway was maintained at a speed of 226 rpm. The XA/210Y analytical balances (Radwag, Radom, Poland) with an accuracy of 0.01 mg were utilized to determine the weight loss of the samples.

X-ray diffraction analysis was performed in Bragg–Brentano geometry (θ/2θ) using the high-resolution diffractometer (Empyrean, Malvern Panalytical, Malvern, UK). The XRD patterns were measured using the Cu Kα (λ = 1.5418 Å) radiation of a tube, operated with a generator voltage of 40 kV and a current of 30 mA. The K-Beta Ni filter was subsequently applied. The data were collected over the range of 2θ = 20 ÷ 100°, with a step size of 2θ = 0.01° and a counting time of 6 s per datum, using the proportional detector. A fixed divergence slit of 1° was used together with a beam mask of 5 mm, and all scans were carried out in continuous mode. The incident and receiving Soller slits were 0.04 rad, and the receiving anti-scatter slit was fixed at 1°. The crystalline phase in the samples was identified using the High Score Plus software package (version 3.0e, Malvern Panalitical, Malvern, UK).

## 3. Results and Discussion

**Figure 4:**
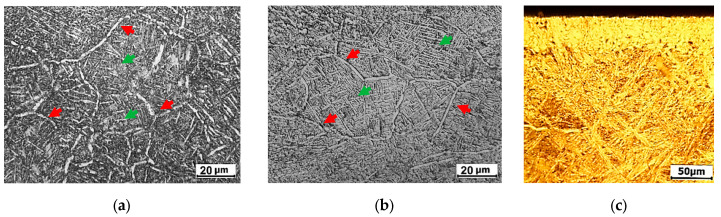
The microstructure of the studied sample. (**a**)—untreated alloy BT22, (**b**)—microstructure of laser point before nitriding, (**c**)—laser point after nitriding.

Microstructure. Figure 4a,b presents the microstructure of the studied alloy samples. It consists of a primary α-phase on grain boundaries and a secondary α-phase formed due to β-α phase transformation during precipitation heat treatment and laser processing. The grain itself represents the α+β colony [38], where the space between α particles is filled with retained β-phase. Grain boundary particles visualize grains and allow grain size measuring.

Figure 4a shows the alloy in as-supplied condition. The bigger 15–60 µm long grain boundary [39] particles are pointed out by red arrows, and finer 5–15 µm particles (green arrows) are inside the grain. The β-grains are equiaxial and have a size of 40–60 µm. The thickness of the grain-boundary α-phase is 1–4 µm, and the thickness of in-grain particles is about 1 µm. This is an expected microstructure for the BT22 alloy in the as-supplied condition [16]. The content of the α-phase nearly equaled the quantity of the β-phase (the β-phase content was 45–52%).

Laser processing slightly changes the structure of the alloy (see Figure 4b). Namely, the grains experienced growth (50–80 µm) due to alloy heating above the α-β transus temperature. There was also thinning and an increase in the amount of the α-phase. The morphology of the α-phase changed from lamellar to the one where the needles cross each other, resembling the structure of martensite [40,41,42,43]. This microstructure was formed due to rapid cooling during the laser processing. The higher supercooling rate of the BT22 alloy after laser treatment is because only a thin layer accepts the energy, and when the laser shuts down, the heat transfers to the lower material layers. The proportion of the β-phase content to that of the α-phase remained unchanged (β-phase ≈ 45–52%).

Figure 4c shows the microstructure of the nitrided alloy in the center of the laser point. The thickness of the compound layer is ~45 µm. The amount of the α-phase significantly increased; determining the ratio of the phases and the size of the β-phase through the metallographic method was very difficult.

Microhardness. Due to the edge effect, the hardness was measured in two ways: on the normal section, 9 µm below the surface (HV_0.05_), and in the second way, by indents on the top of the sample (HV_0.01_).

**Figure 5:**
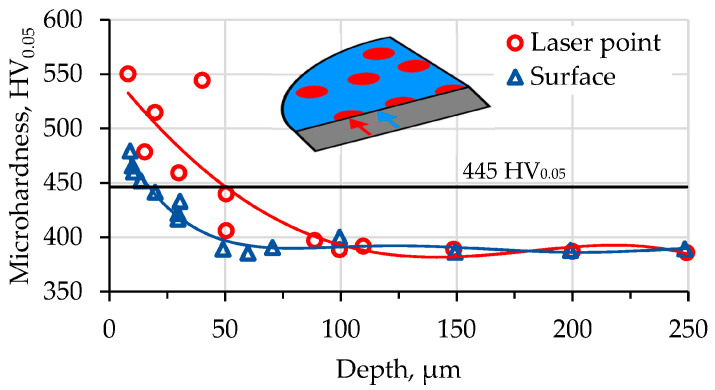
Hardness profile of laser-processed and nitrided BT22 alloy.

According to the hardness profile of the laser+nitride alloy, the depth of the saturation layer is 15–20 µm for the surface (in the middle, between the laser points). The maximum subsurface hardness here was 460–480 HV_0.05_ (see Figure 5). The microhardness measurements indicated that the thickness of the thermodiffusion layer below the laser spot was 45–50 µm. As a margin for the nitride layer, we considered the line where the hardness was 15% higher than it was initially (445 HV_0.05_). The microhardness measured in the center of the laser point at a depth of 9 µm was 580–595 HV_0.05_, which is 20% higher than that between the points. The sample that was only nitrided had the same hardness and diffusion layer thickness as the material between the points. Additionally, three indentations were made in order to measure the surface hardness on the top of each sample and to determine the maximum surface hardness. We put indentations on the surface using the lowest load available for the device: 10 g (0.098 N). At the center of the laser point, the microhardness was 1190 ± 45 HV_0.01_, and in the space between the points, it was 1080 ± 39 HV_0.01_. These values are quite big. They are more than those obtained for the titanium alloy Ti5Al4V2Mo (600–800 HV_0.05_) [43]. Smaller values were obtained at a depth of 10 µm for the BT22 alloy in this study. In other studies, harder surfaces have been obtained: 1550 HV_0.05_ in [44] or 2500 HV_0.05_ in [45]. The bigger surface hardness achieved in the current research was 1190 HV_0.01_, which is more than that found for the method described in [46], roughly the same as that in [45] (1550 HV_0.1_) and [44] (1000–1300 HV_0.1_), but less than that found in [46]. The depth of the nitrided layer obtained in this study was close to the values reported in [24,47,48] (15–50 µm), but less than 120 µm, as found in [49].

**Figure 6:**
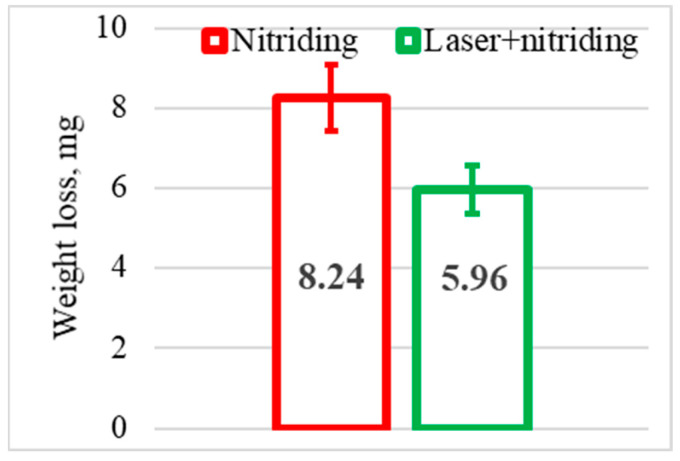
Wear loss of tested specimens.

Wear-Resistance. The average weight loss of the samples is illustrated in Figure 6. It indicates that the wear-resistance of the laser+nitrided sample is 28% higher: 5.96 mg vs. 8.24 mg. This is because the nitrided layer separates the friction surfaces created by a natural or friction-induced oxide film [50], thus preventing contact between the two metals.

**Figure 7:**
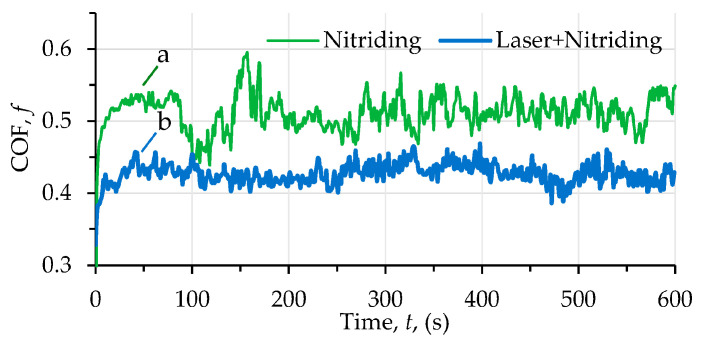
Friction factor diagram for the studied BT22 alloy: (**a**)—nitriding, (**b**)—laser treatment + nitriding.

The changes in the friction coefficient of the nitrided and laser+nitrided alloys (based on four tests) are presented in Figure 7. In contrast to the nitride alloy, the laser-treated one has a more stable CoF graph, is smoother, and is also free of an initial rapid increase. We suggest that the fine structure of the laser dots resulting in fine wear debris more effectively separates the friction surfaces. A smaller particle size also produces less abrasive damage. The average CoF value for the nitride alloy is 0.51, while it is 0.43 for the other, which is 16% less.

**Figure 8:**
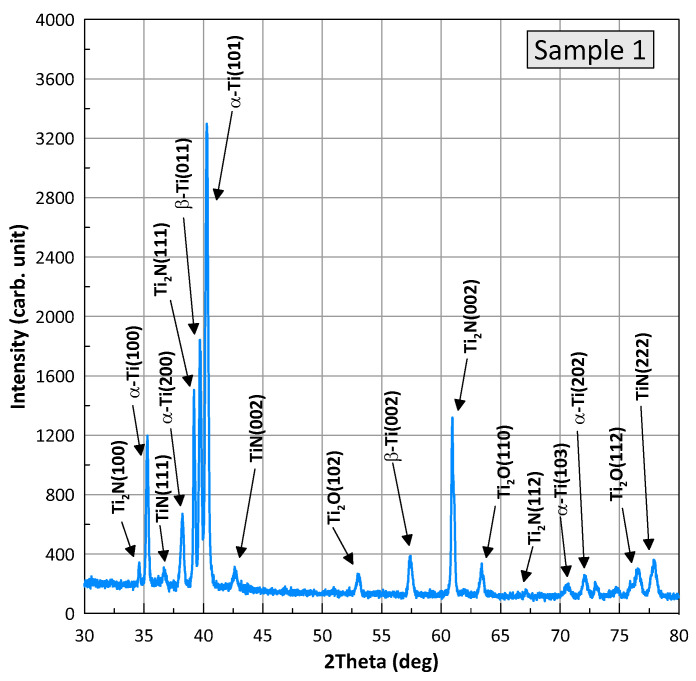
X-ray diffraction profiles for the nitrided sample (sample 1).

**Figure 9:**
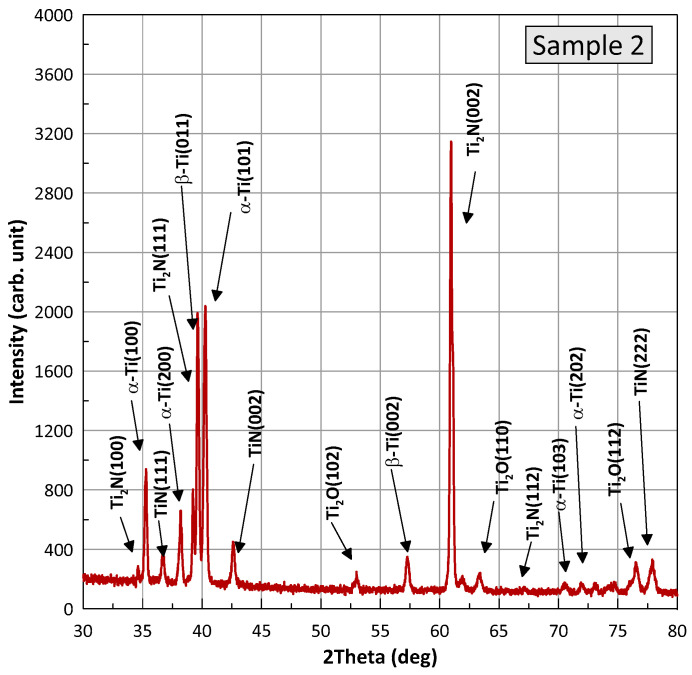
X-ray diffraction profiles for the laser-processed and nitrided sample (sample 2).

Phase identification. Figure 8 and Figure 9 show XRD patterns for samples of the BT22 titanium alloy after the wear test, referred to as samples 1 (nitriding) and 2 (laser+nitriding). The presented profiles for the samples have the same X-ray diffraction peaks, differing relative intensities, and slight position shifts. Phase analysis was performed with HighScore Plus v. 3.0e and using the Crystallography Open Database. It was found that for both samples, the positions of the peaks correspond to four phases present: alpha-Ti with a hexagonal structure and the space group P63/mmc (Card No.: COD 96-900-8518), TiN with a cubic structure and the space group F m-3m (Card No.: COD 96-101-1103), Ti_2_N with a tetragonal structure and the space group P 42/m n m (Card No.: COD 96-110-0032), Ti_2_O with a hexagonal structure and the space group P-3m1 (Card No.: COD 96-153-2774), and a small amount of β-Ti with a cubic structure. The individual phases are marked at the diffraction peaks in the figures. An analysis of the width of the diffraction half-peaks shows that the grain sizes for Ti are in the range of 350–450 nm, for Ti_2_N in the range of 700–800 nm, and for Ti_2_O in the range of 150–230 nm. The performed Rietveld analysis allowed for the determination of the proportion of the individual phases. Detailed data are summarized in Table 2.

**Figure 10:**
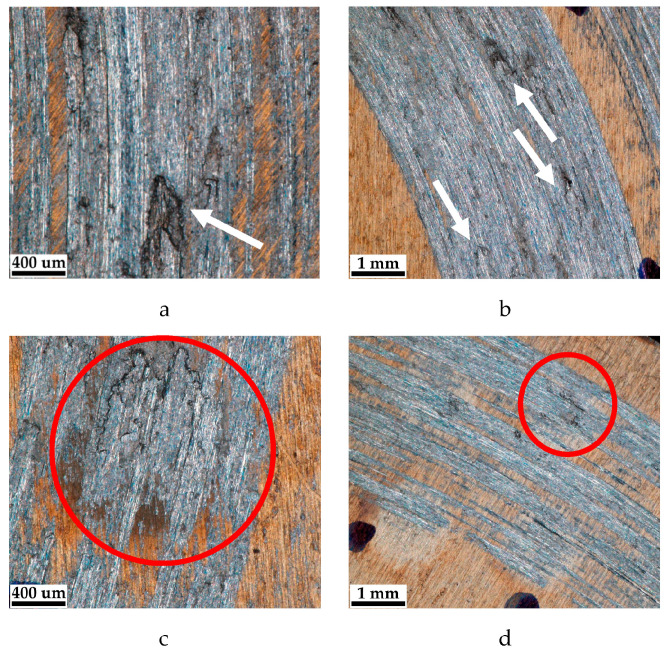
Optical micrographs of the samples’ friction surface: (**a**,**b**)—nitrided alloy, (**c**,**d**)—alloy nitrided after selective pulse laser processing.

Surface characterization and wear mechanism. The surfaces of both alloy samples were entirely covered with a yellow film. In Figure 10a,b, white arrows point to the sites where 300–400 µm particles separate from the surface. This is the evident reason for the lower wear-resistance of the nitrided alloy as compared to the one after combined laser-thermochemical processing.

In Figure 10c,d, the laser spot has a diameter of 2.5 mm. Two areas are revealed: the darker parts, with a diameter of 500–700 µm, and the halo around them, which is the heat-affected zone. We point to the fact that the surface of the laser spot in Figure 10c,d are the places where worn material has accumulated (inside the red circles). In our opinion, this is a reason for the increased roughness of the friction surface. These sites likely contribute to the separation of the disc and pin, and thus reduce both the coefficient of friction and wear.

**Figure 11:**
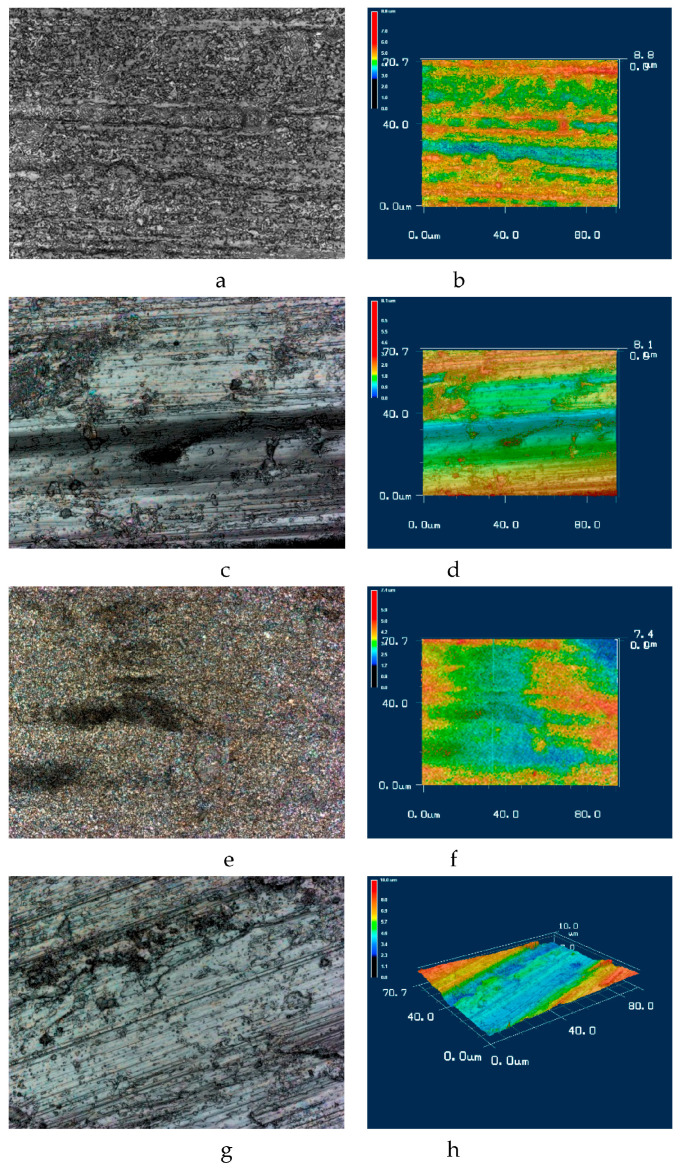
Surface topography of the tested samples: (**a**,**b**)—intact surface and the profile of the intact surface of nitrided alloy; (**c**,**d**)—friction surface and the profile of nitrided alloy; (**e**,**f**)—friction surface and the profile of nitride laser point; (**g**,**h**)—friction surface and the profile of the laser-processed nitrided alloy. Corresponding Ra and Rz values are in Table 3.

The images in Figure 11a–h and colored topography maps were scanned by a laser confocal microscope. The results of the roughness measurement are presented in Table 3. The magnification of the images is 50, and the scale may be derived from the 3D profiles. The roughness of the nitrided alloy surface is less than the roughness of combined-processed sample between the laser spots. The roughness of the nitrided laser point is less than that of the nitrided surface. After the friction test, the roughness of the laser+nitrided alloy is higher than that of the nitrided alloy.

**Figure 12:**
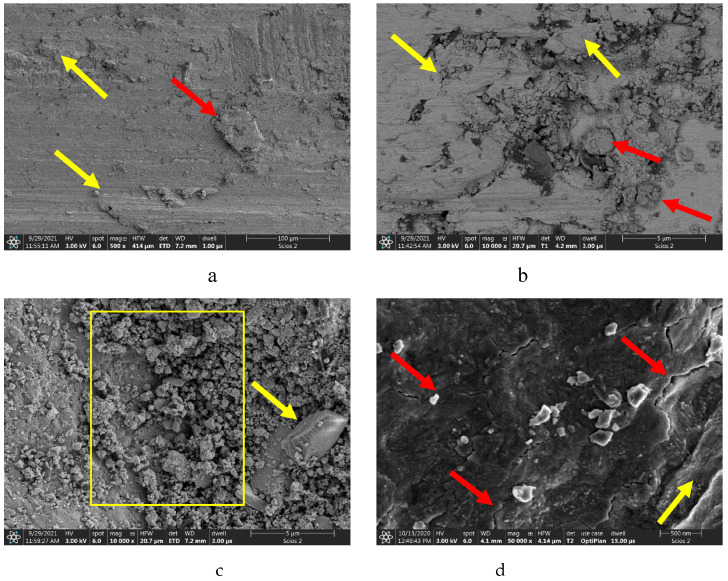
Friction surface of sample 1: (**a**)—metal flows on the surface (yellow arrows) and torn-off metal particles (red arrow); (**b**)—yellow arrows point plastic deformations and surface cracks, red arrows point wear particles; (**c**)—agglomeration of worn particles on the friction surface, arrow points coarse particle causing abrasive wear; (**d**)—microcracks on the friction surface (red arrows) and surface ploughing (yellow arrow).

**Figure 13:**
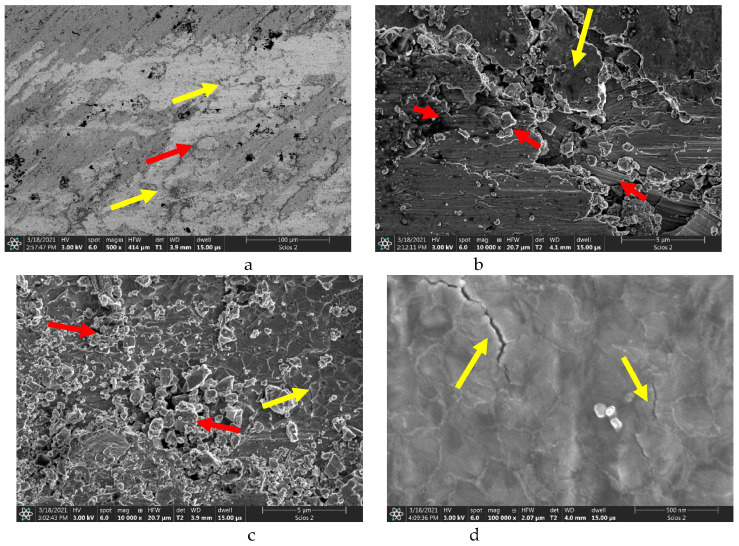
Friction surface of sample 2. (**a**)—minor delamination of friction surface (yellow arrow) and torn-off particle (red arrow); (**b**)—submicron scratches on the surface (red arrows), wear particles (yellow arrow), (**c**)—wear particles (red arrow) and surface nano-texture in the center of laser spot; (**d**)—nano-cellular microstructure formed on the surface after selective laser treatment and nitriding. Yellow arrows point small microcracks on the surface.

The SEM images of the friction surfaces in Figure 12 represent the nitrided sample 1, and those in Figure 13 represent the laser-treated and nitrided sample 2. On the surface of sample 1, we detected metal flows and delaminations, which can be clearly seen in Figure 12a. Their dimensions are more than 300–400 μm. The red-arrowed particles, 30–50 μm in size, possibly arise due to the tear-off of delaminated portions of the material. At higher magnification, in Figure 12b, the places of surface plastic deformation and cracking may be observed. Red arrows point to wear products formed as a result of surface cracking. The size of the worn particles may be less than 1–5 μm. Figure 12c shows the place with the accumulation of worn particles. The smaller particles are about 1 μm and rounded, while the bigger particles are about 5 μm and have sharp edges (arrowed). In the left corner of the yellow box, beneath the wear particles, some fine cracks are clearly visible. As observed closer (Figure 12d, red arrows), they have a thickness of few tens of nanometers. These cracks are the reason for the formation of fine near-micron-size wear particles, while bigger ones are formed due to the tear of delaminated metal. The red arrow shows the ploughing of the surface by fragmented nitride particles.

Figure 13 shows the friction surface of sample 2. Here we also detected metal flows (Figure 13a), but their size is much lower than that in Figure 12a, and the delaminated wear particle (red arrow) is not bigger than 20 μm. Thus, delamination makes an insignificant impart to the wear mechanism. The area around the yellow arrow is a layer transferred from the counterpart; it is lighter than the general nitrided surface, thus containing fewer light elements. At a bigger magnification, in Figure 13b, it may be observed that bigger wear particles are formed as a result of tearing (yellow arrow), and smaller ones have a size less than 1 μm. At the laser point, the surface is smooth and contains submicron wear particles (red arrows, Figure 13c), which are less than the expected clearance between the disc and the pin. A distinct cellular microstructure is pointed to by the yellow arrow. The size of these cells is only 300–400 nm, and some of them (arrowed, Figure 13d) are only 40–100 nm. At the same magnification (Figure 12d and Figure 13d), sample 1 has no nano-cells, but only cracks on the surface. Therefore, the nano-cellular microstructure was formed as a result of the proposed treatment—laser+nitriding—because no cells were found on the surface of sample 1 and beside the laser point on sample 2.

The proposed combined treatment, which consists of selective laser treatment and diffusion nitriding, created a heterogeneous microstructure, consisting of a general surface and strengthened laser-treated spots. Fast heating and cooling around the phase transition point promoted the growth of β-grains and the simultaneous refinement of the α-phase (Figure 4a,b). The α/β ratio remained unchanged. The diffusion of interstitial atoms into titanium is easier along the α-phase boundaries [15,16,17,18]. Due to the refinement of α-phase, the number of diffusion passes increased and the depth of N diffusion increased, which could be identified through the 20% increased hardness at the laser point (Figure 5). So, the material in the laser spot is harder, and the thickness of the diffusion layer is also bigger. Thus, a composite-like surface structure was formed, where harder “phases” (laser spots) were embedded in a softer “matrix”. The roughness of the laser point was reduced as compared to the surrounding area. We suppose that during selective laser treatment the surface micro-asperities flatten. Additionally, the other reason is the refinement of the α-phase.

Tribological tests indicated that sample 2 had a 16% lower coefficient of friction and a 28% lower wear weight loss (Figure 6 and Figure 7). As for sample 1, its surface roughness after friction was less than that of sample 2, but its wear-resistance was less. This may be explained by the lower amount of titanium oxides and the lower concentration of nitrides (Figure 8 and Figure 9, Table 3) as well as a less rigid surface due to less thickness of the diffusion layer. Consequently, the material wears more actively. The wear products formed were coarser and contributed to abrasive wear. In Figure 12a,b, bottom section, and especially in Figure 11c,d, there is clear evidence of abrasive wear. The depth of the blue scratch is more than 1 μm. The friction surface of sample 2 also has micro-scratches (Figure 11g,h). Hence, micro-abrasive wear was observed for both samples, but for the nitrided one it was much more intensive. As it may be seen from Figure 12a, big wear particles are formed due to the tearing of delaminated portions of the nitrided surface and deformed surface areas (Figure 12b). Smaller particles also form and also contribute to the wear process. They are formed due to surface fracture and cause abrasive damage to it. The big number of rounded worn particles on the surface of sample 1 indicates that they had been wedging between the disc and the pin for a long time. Additionally, they should be quite ductile. In Figure 12d, yellow arrow, and Figure 11a,c, the surface is also significantly damaged by scratching, which is the evidence of abrasive wear. To end, the wear track is covered by a dense network of microcracks, producing new portions of abrasive particles. Thus, we supposed that for this sample, the dominant wear mechanism is delamination and micro-abrasive wear.

Sample 2, which was laser-processed and then nitrided in identical conditions to those of sample 1, had better tribological performance. We noted earlier (Figure 10c,d) that some of the harder laser-treated spots actively collected wear products, and with time, they started to lift off the surface, which may have caused the increased values for the friction surface roughness. Abrasive particles clamped on to the surface with lesser force and therefore caused less abrasive action. This reduced the coefficient of friction. In addition, it has been reported that titanium oxide may behave as a solid lubricant and thus reduce the coefficient of friction. Indeed, according to Figure 8 and Figure 9 and Table 2, sample 2 contains 24% of T_2_O (24% vs. 17.6%). The wear products in sample 2 were smaller and the surface delamination was observed to a much less extent. Therefore, they did not wedge for long between the friction partners. At the same time, Figure 13b shows where the surface micro-delamination sites were found. The size of torn-off particles is only about 1–5 μm. They are located on the laser spots and represent the material transferred from the counterpart (as-supplied alloy BT22, Figure 13a). Beneath them (right-bottom corner, Figure 13b–d), the smooth sample surface is slightly damaged by abrasion. The nano-cellular microstructure (in contrast to the micro-cellular [33]) discovered on the surface of the laser points was smooth and only slightly damaged. The microchannels formed on the surface did not contribute a lot to the formation of the wear products; however, in the case of the use of lubrication, they may effectively retain the oil. This will definitely increase the tribological output of the material. Thus, for sample 2 (selective laser treatment + nitriding), the leading wear mechanism was micro-abrasive wear.

## 4. Conclusions

The proposed technology of the combined surface treatment of two-phase titanium alloys may be used with a minor increase in cost, and it may also be carried out during the common process of the two-step aging of the alloy. The selective laser treatment with a power density of 0.4 GW/cm^2^ refined the grains of the α-phase and facilitated nitrogen diffusion, resulting in the formation of a nano-cellular microstructure on the surface after nitriding.

The combined surface treatment increased surface hardness in the place of focus (1190 HV_0.01_ vs. 1080 HV_0.01_). At a depth of 9 μm, the microhardness levels achieved were 580–595 HV_0.05_ and 460–480 HV_0.05_, respectively. The thickness of the thermodiffusion layer was 15–20 μm between the laser spots, and 50 μm below the laser spots.

The alloy treated according to the proposed method had a 16% lower coefficient of friction with a smoother graph, and a 28% lower weight loss in dry sliding conditions.

The strong nano-cellular microstructure at the laser point improved the tribological performance of the alloy, promoted a reduction in the size of the wear particles, and was efficiently resistant to scratching. The microchannels formed on its surface, and the nano-cells themselves may effectively retain oil in the case of lubricated friction.

The dominant wear mechanism for the nitrided sample was micro-abrasive wear, and that for the selectively laser-treated and nitrided sample was micro-abrasive wear accompanied by delamination.

## Figures and Tables

**Table 1:** Chemical composition of titanium alloy BT22.

Alloy	Al	V	Mo	Cr	Fe	Zr	General Properties
BT22	5.2	4.8	4.8	1.2	1	<0.3	UTS 1100–1400 MPa, good machinability, and weldability, elongation 10%. α-β transition point ~830 °C. the α-β ratio in annealed condition: ~50/50 [14].

**Table 2:** The proportion of individual phases in BT22 titanium alloys.

Sample No.	α-Ti (%)	Ti_2_N (%)	TiN (%)	Ti_2_O (%)	β-Ti (%)
1	53.9	17.7	6.9	17.6	3.9
2	43.0	16.0	9.0	24.0	8.0

**Table 3:** The roughness of the samples from Figure 11.

Sample	Location	Parameter	
		Rz, μm	Ra, μm
1	Nitrided Surface	2.81	0.36
	Wear track	4.13	0.44
2	Nitrided Surface	4.32	0.53
	Laser point	2.23	0.19
	Wear track	4.56	1.08

## Data Availability

The research data are available on request. Please, contact corresponding author.
